# Kinetics and Optimization of the Photocatalytic Reduction of Nitrobenzene

**DOI:** 10.3389/fchem.2019.00289

**Published:** 2019-04-24

**Authors:** Julia Patzsch, Benedict Berg, Jonathan Z. Bloh

**Affiliations:** DECHEMA-Forschungsinstitut, Chemical Technology Group, Frankfurt, Germany

**Keywords:** photocatalysis, titanium dioxide, nitrobenzene, aniline, kinetics, optimization

## Abstract

The photocatalytic reduction of nitrobenzene to aniline in alcoholic solutions appears as an interesting alternative to the classical hydration. However, little is known about the influence of reaction parameters on the kinetics of the reaction which were therefore studied herein. The effects of light intensity, catalyst concentration, initial concentration, and temperature were systematically investigated under more than 50 different conditions and accurately described with an appropriate kinetic model. The results show that the efficiency of the reaction is extremely high and apparent quantum yields of up to 142 % were observed under optimized conditions. Particularly interesting is the fact high efficiencies were also obtained at high reaction rates of up to 74.3 mM h^−1^. Overall these results demonstrate that heterogeneous photocatalytic reactions can be very efficient and productive at the same time and may therefore present a powerful tool in synthetic organic chemistry.

## 1. Introduction

With an annual world production of about 4 Mt, Aniline is one of the 100 most important building blocks in the chemical industry (Kahl et al., 2011). It is used as a precursor for polymers (mainly polyurethanes), rubber additives, dyes, herbicides, and pharmaceuticals (Kahl et al., 2011). Today, aniline is predominantly produced by catalytic hydrogenation of nitrobenzene (Kahl et al., 2011). This is a very exothermic process that has a large carbon footprint due to the need for three equivalents of hydrogen gas. Moreover, the corresponding oxidation reaction only yields essentially valueless (waste) water. Therefore, more environmentally friendly alternative routes are highly sought-after. The holy grail in this context, the direct amination of benzene, has so far only yielded mediocre results due to the need for high ammonia excess (Becker et al., 2000; Yu et al., 2014). However, there exist several other promising alternatives, amongst them the photocatalytic reduction of nitrobenzene in alcohols.

Photocatalysis with semiconductor nanoparticles can be used in organic synthesis to catalyze reactions such as oxidation and reduction reactions, C-C bond formation or cyclizations (Friedmann et al., 2016; Bloh and Marschall, 2017). Once photons with sufficient energy are absorbed by the photocatalyst, electrons are excited from the valence to the conduction band. The so generated electron-hole-pairs can subsequently induce chemical reactions (Schneider et al., 2014). In this particular case, the conduction band electrons reduce nitrobenzene to anilin in six consecutive one-electron transfer reactions (Flores et al., 2007). At the same time, the alcohol is oxidized to the corresponding aldehyde. As this only yields two electrons, the stoichiometry requires three equivalents of the alcohol. This reaction shows a considerable substrate scope, both with respect to the nitroarene as well as the alcohol (Selvam and Swaminathan, 2011b; Hakki et al., 2013b). Moreover, if suitable reaction partners are chosen, the generated primary products may undergo further reactions to N-alkyl- or quinoline derivatives which offers an interesting direct synthetic pathway to those more complex molecules (Hakki et al., 2013a,b; Hirakawa et al., 2015). These follow-up reactions are purely acid-catalyzed though and non-photoinduced.

However, despite their many advantages such as high energy input at mild reaction conditions, the use of less energetic substrates and the reduced formation of by-products, photocatalytic reactions are currently of little importance in the chemical industry. The main obstacles for a more widespread use are limited availability of standardized and scalable technical equipment for the reactions and little knowledge of the reaction mechanisms and kinetics which make optimization and scaling up extremely challenging (Bloh and Marschall, 2017). Therefore, the reaction kinetics as well as the influence of the reaction parameters on the photocatalytic reduction of nitrobenzene are studied in detail herein, in an attempt to optimize the reaction both with respect to efficiency and productivity to pave the way for an industrial implementation. The studied reaction is the nitrobenzene reduction in ethanol that also yields the above-mentioned condensated products such as quinolines (Hakki et al., 2013a,b; Hirakawa et al., 2015). However, while the overall aim is to eventually understand the kinetics of the whole reaction pathway up to those quinolines, this particular study focuses only on the first part of the cascade, the photocatalytic reduction of nitrobenzene to aniline with simultaneous oxidation of ethanol to acetaldehyde.

## 2. Experimental

The experiments were done in the photoLAB Batch-S system (Peschl Ultraviolet) equipped with four identical reactors with a volume of 25 mL each that are arranged around a 20 W (radiant flux) TQLED100.10/365 lamp equipped with 365 nm LEDs. The temperature in the glass reactors is controlled by circulating water through an outer jacket. The light intensity (given as volumetric photon flux density *q*_*p*_) was varied from 4μmolL^−1^s^−1^ to 100μmolL^−1^s^−1^ and verified by ferrioxalate actinometry (Hatchard and Parker, 1956). In a typical experiment, the TiO_2_ catalyst (0.25 g L^−1^ to 12.5 g L^−1^, Aeroxide P25, Evonik) was suspended in 20 mL absolute ethanol (SeccoSolv, Merck) containing 10 mmol L^−1^ nitrobenzene (Acros). The irradiation was started after 30 min purging with Argon to ensure an oxygen free system. The temperature was adjusted to 25 °C and the mixture continuously stirred. Samples were taken during the reaction and analyzed by GC after removing the particles through filtration (0.2 μm PTFE) from the reaction mixture.

For the analysis of all substances except for acetaldehyde a Thermo Trace GC Ultra with a FID detector equipped with a 30 m FS-Supreme-5 ms (0.25 mm diameter) capillary column was used. Operating parameters: injection temperature 250 °C, oven temperature 70 °C (hold 2 min) from 70 °C to 170 °C at the rate of 10 K min^−1^, from 170 °C to 320 °C (hold 1 min) at a rate of 40 K min^−1^ in splitless mode, injection volume (1 μL) with nitrogen as carrier gas. Acetaldehyde was analyzed with a Thermo Focus GC equipped with a FID detector and a 25 m CP-Chirasil-DEX CB (0.25 mm diameter). Operating parameters: injection temperature 250 °C, oven temperature 41 °C, from 41 °C to 43 °C at a rate of 0.5 K min^−1^, from 43 °C to 200 °C (hold 9.5 min) at a rate of 80 K min^−1^ in split mode (10 mL min^−1^), injection volume (1 μL) with nitrogen as carrier gas. The concentration of the substances was calculated from the peak areas via calibration with authentic standards. In case of ethylideneaniline an authentic standard was not available, therefore the concentration was estimated using the calibration factor of aniline and correcting for the number of carbon atoms.

## 3. Results and Discussion

In order to better understand the kinetics and the influence of reaction parameters, the photocatalytic reduction of nitrobenzene in ethanol using TiO_2_ as a photocatalyst was studied under a variety of different conditions, with the light intensity (4μmolL^−1^s^−1^ to 100μmolL^−1^s^−1^) and catalyst concentration (0.25 g L^−1^ to 12.5 g L^−1^) each spanning about 2 orders of magnitude. Additionally, the temperature was varied from 15 °C to 65 °C. In total, this encompassed experiments under 50 different conditions, excluding duplicates.

**Figure 1:**
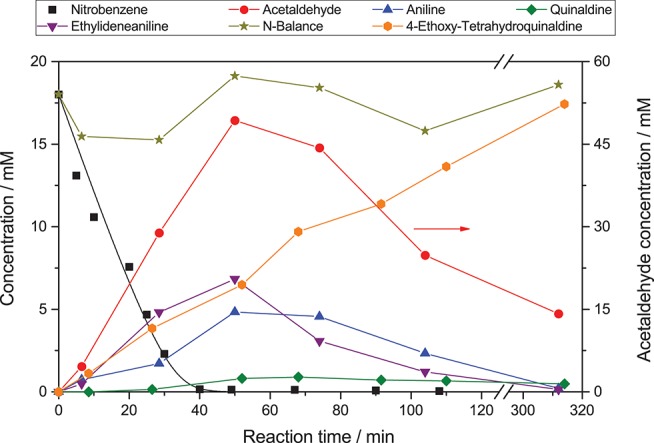
Exemplary time course of the photocatalytic conversion of nitrobenzene (reaction conditions: 20 mM nitrobenzene and 2.5 g L^−1^ TiO_2_ in 20 mL ethanol with 0.26 mM tosylic acid, *q*_*p*_ = 100 µM s^−1^, 25 °C).

Figure 1 shows an exemplary time course of this reaction. At first, nitrobenzene (**1**) is reduced in three consecutive reactions to aniline (**4**), via nitrosobenzene (**2**) and phenylhydroxylamine (**3**). These intermediates are qualitatively observed but not quantified due to their low concentration. Each of those reactions is a two-electron transfer, however, these presumably happen in consecutive one-electron transfer events via a radical intermediate (Flores et al., 2007). Simultaneously, ethanol (**5**) is oxidized to acetaldehyde (**6**) in two consecutive one-electron transfer reactions, via an alcohol radical (Schneider and Bahnemann, 2013; Burek et al., 2019b). The stoichiometry of the reaction demands that three equivalents of ethanol are oxidized to supply the electrons for complete nitrobenzene reduction. After 40 min of irradiation, nitrobenzene is fully converted. After this time the color of the suspension changed from white to light blue, presumably a consequence of the well-known partial reduction of the TiO_2_ surface by trapping of photogenerated electrons as Ti(III), since an electron acceptor is no longer available (Mohamed et al., 2011).

The reaction does not stop at aniline and acetaldehyde and follow-up condensation products are readily observable. These reactions are acid-catalyzed and while they proceed over TiO_2_ as a result of its acidic surface groups, they are much faster in the presence of an added acid (Hakki et al., 2013a). In order to better illustrate these reactions, the example given in Figure 1 was therefore done in the presence of a small amount (0.26 mM) of added tosylic acid. In the first step, acetaldehyde condenses with aniline to ethylideneaniline (**10**). Subsequently, a Povarov reaction of ethylvinylether (**11**, formed from acetaldehyde and ethanol) with ethylidenaniline leads to 4-ethoxy-1,2,3,4-tetrahydroquinaldine (**12**) (Park et al., 1995). This can eliminate ethanol to form 1,2-dihydroquinaldine (**13**) and subsequent oxidation, reduction or disproportionation lead to quinaldine (**14**) and 1,2,3,4-tetrahydroquinaldine (**15**). All of these intermediates have been previously reported and were identified using GC-MS as well as verified and quantified using GC-FID (Park et al., 1995; Selvam and Swaminathan, 2012; Hakki et al., 2013a).

**Figure 2:**
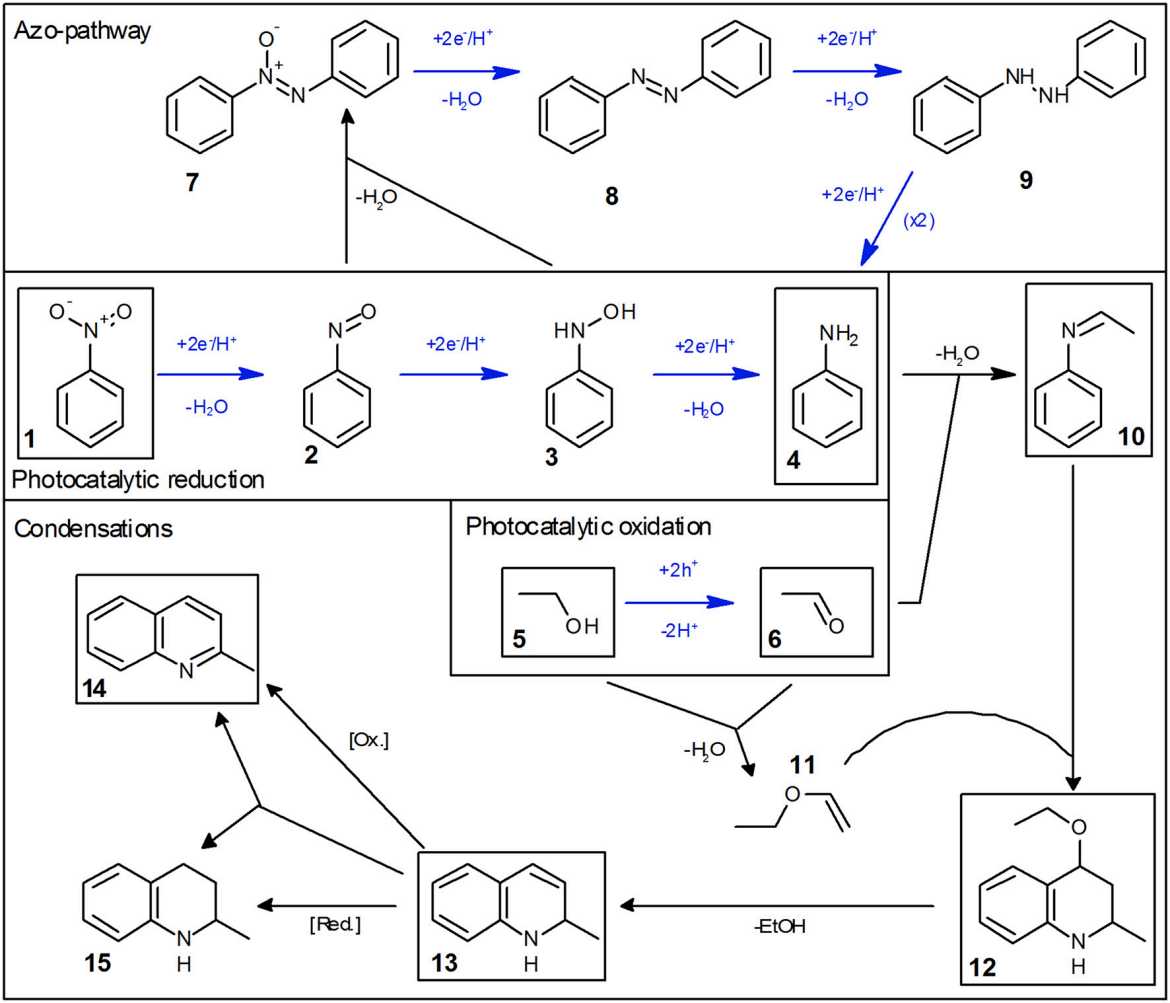
Mechanism of the photocatalytic reduction of nitrobenzene in ethanol. Photocatalytic reaction steps are marked with blue arrows and all major intermediates/products are marked with a black box.

Some authors report the formation of quinaldine as the major product. This was not observed here, probably since the equilibrium is pushed toward 4-ethoxy-1,2,3,4-tetrahydroquinaldine by the high ethanol concentration (Selvam and Swaminathan, 2012; Hakki et al., 2013a). However, the authors note that conversion of 4-ethoxy-1,2,3,4-tetrahydroquinaldine to quinaldine readily occurs during injection into the GC if the temperature is too high and may therefore falsely detect as quinaldine. The complete reaction scheme is summarized in Figure 2.

The intriguing property of the reaction to form condensated products is currently explored as a means to directly access these complex molecules in a one-pot reaction but will not be studied in detail here (Hakki et al., 2013a,b; Hirakawa et al., 2015). The nitrogen-balance remains virtually constant throughout the reaction, indicating that all major intermediates and products are accounted for. The small variations observed are attributed to adsorption on the photocatalyst particles and experimental error of the GC, particularly since not all of the intermediates were available as standards and calibration factors had to be estimated. After about 300 min the reaction is fully complete and the nitrobenzene is converted almost quantitatively to 4-ethoxy-1,2,3,4-tetrahydroquinaldine (94 %) with traces of quinaldine (3 %), 1,2-dihydroquinaldine (2 %), and aniline (1 %). Since the condensation reactions are purely acid-catalyzed and non-photoinduced (switching off the light after 40 min does not have a significant effect) they are ignored in the present study as the focus is on the rate of photocatalytic reduction of nitrobenzene. As the nitrogen balance is complete and all products observed are formed out of aniline, the reduction rate of nitrobenzene also equals the formation rate of aniline. Also, all of the subsequently reported reactions were done without the addition of an acid since the condensation reactions were not of interest.

The reduction rate of nitrobenzene was generally observed to be pseudo zero-order at higher concentrations, below 1 mM it gradually leveled off into first-order. This fits very well with a substrate limitation, since the adsorption constant of nitrobenzene on TiO_2_ was reported to be >1 mM^−1^ (Makarova et al., 2000; Tada et al., 2004). While attempts to fit the concentration profiles using numerical simulation of the Langmuir-Hinshelwood kinetic expression (*cf*. Figure 1, black line) yielded good results, it has been highlighted several times that Langmuir-Hinshelwood is not an appropriate kinetic model for photocatalytic reactions (Ollis, 2018; Muñoz-Batista et al., 2019). As analysis of the mixed-order regime at lower nitrobenzene concentrations later in the reaction may be influenced by kinetic disguises, (Ollis, 2018) here only the initial zero-order reaction rate (*k*_*nb*_) was determined using linear regression. These zero-order reduction rates were subsequently modeled and analyzed using a recently published kinetic model (*vide infra*) (Bloh, 2019; Burek et al., 2019a).

**Figure 3:**
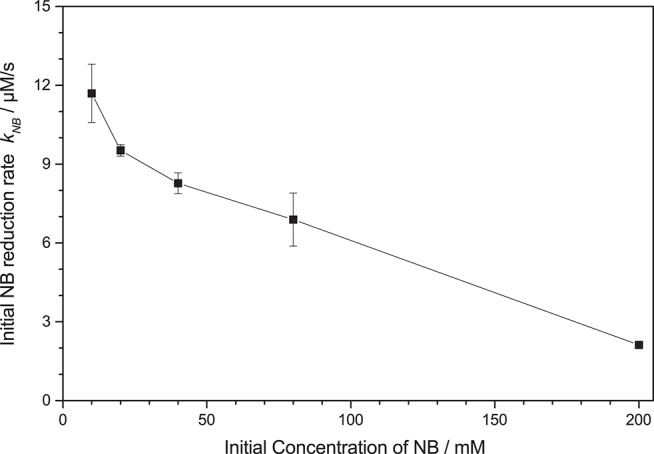
Dependence of the initial nitrobenzene reduction rate *k*_*nb*_ on the initial nitrobenzene (NB) concentration (reaction conditions: 2.5 g L^−1^ TiO_2_ in 20 mL Ethanol, *q*_*p*_ = 100 µM s^−1^, 25 °C).

Initial studies have shown that the photocatalytic reduction rate is reduced significantly at higher concentrations of nitrobenzene, *cf*. Figure 3. This can readily be explained by studying the mechanism of classical nitrobenzene hydrogenation with hydrogen gas. This process is also run only at low nitrobenzene concentrations, since the intermediately formed nitrosobenzene (**2**) and phenylhydroxylamine (**3**) are quite reactive and can readily condense with other intermediates or aniline to form azoxybenzene (**7**) or azobenzene (**8**), *cf*. Figure 2, “Azo-pathway” (Maldotti et al., 2000; Gelder et al., 2005). In fact, traces of these compounds could be identified in the GC-MS data. While these may also be further reduced to eventually yield aniline, their reduction rate is typically much slower, which would also slow down the overall observed reaction rate (Maldotti et al., 2000; Gelder et al., 2005). Additionally, these compounds are strongly colored and may therefore decrease the photocatalyst efficiency by reducing the photon flux available to it (parasitic light absorption). As this side-reaction is a bimolecular reaction following second-order kinetics, the probability for it increases exponentially with the concentration of the intermediates and thereby in turn, with the nitrobenzene concentration (Maldotti et al., 2000; Gelder et al., 2005). Since below a nitrobenzene concentration of 10 mM, the reaction rate was not further increased, all the following studies were performed at initial concentrations of 10 mM. Apparently, at this concentration, the azo-pathway is so strongly suppressed that is has no measurable impact on the overall reaction rate.

**Figure 4:**
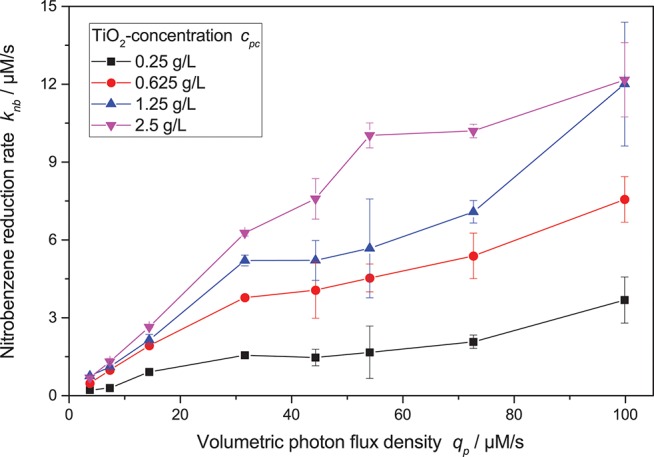
Dependence of the nitrobenzene reduction rate *k*_*nb*_ on the volumetric photon flux density *q*_*p*_ for different photocatalyst concentrations (reaction conditions: 10 mM NB concentration, 25 °C).

Figure 4 shows the nitrobenzene reduction rate as a function of the light intensity. At low light intensity, the reaction rate increases approximately linearly. However, at higher light intensity, the reaction rate increasingly shows diminishing returns. This behavior was often observed for other photocatalytic reactions as well and attributed to increased charge carrier recombination at higher light intensity (Upadhya and Ollis, 1998; Satuf et al., 2008; Valencia et al., 2011; Mills et al., 2015; Nosaka and Nosaka, 2018). Some kinetic models account for this by using a mixed linear and square root dependence for the light intensity (Ollis, 2005; Mills et al., 2006; Dillert et al., 2013; Montoya et al., 2014; Camera-Roda et al., 2015; Deng, 2018). Attempts to fit this model to the present data were successful if the photocatalyst concentration was kept constant.

**Figure 5:**
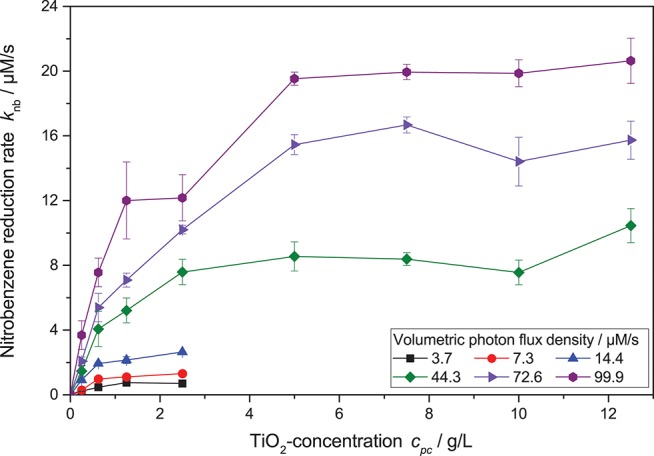
Dependence of the nitrobenzene reduction rate *k*_*nb*_ on the photocatalyst concentration *c*_*pc*_ for different light intensities (reaction conditions: 10 mM NB concentration, 25 °C). A zoomed-in version with only the lower catalyst concentrations can be found in the SI, Figure S4.

However, when also taking different photocatalyst concentrations into account, it became obvious that the inflection point where the reaction order switches from linear to square root dependence on the light intensity also changes with catalyst concentration—the higher the catalyst concentration, the longer the reaction order stays linear. This shift in the catalyst saturation point is even more obvious when the reaction rate is plotted vs. catalyst concentration at different light intensities, *cf*. Figure 5. While for lower light intensities, the reaction rate appears saturated already at catalyst concentrations of 1.25 g L^−1^, it takes about 5 g L^−1^ for the highest studied light intensities. This behavior could not be readily explained by the classical models.

However, we recently published a new model that takes into account the inter-dependency of light intensity and catalyst concentration (Bloh, 2019; Burek et al., 2019a). This model describes the local reaction rate *r* as a function of the intrinsic quantum yield ϕ, kinetic constant k^*^, recombination rate *k*_*r*_, local volumetric rate of photon absorption $L_{p}^{a}$ (LVRPA) and catalyst concentration *c*_*pc*_, Equation 1. With respect to both light intensity and catalyst concentration, a saturation-type dependency is observed. However, increasing one of the parameters also increases the amount of the other parameter needed for saturation. This explains the above mentioned inter-dependency of light intensity and catalyst concentration (Bloh, 2019; Burek et al., 2019a).

(1) $$\begin{array}{r} r=\frac{\phi \cdot L_{p}^{a}\cdot k^{*}\cdot c_{pc}}{\phi \cdot L_{p}^{a}+k_{r}+k^{*}\cdot c_{pc}} \end{array}$$

This reaction rate only models the local reaction rate, to obtain average global rates, it has to be integrated over the whole reaction medium, taking the local light distribution into account. This can be a tedious and very time-consuming task (Camera-Roda et al., 2005; Satuf et al., 2008). However, if in a simplified approach the local light distribution is only modeled to attenuate alongside one axis of the reactor, i.e., unidirectional illumination, integration over the reaction medium yields the explicit Equation 2 (Bloh, 2019; Burek et al., 2019a). Here, the (average) volumetric photon flux density *q*_*p*_ obtained from chemical actinometry can be used and the local light distribution is no longer needed. Since in the present case, the reaction vessel is cylindrical and illuminated from the side, the light path is not uniform across the width of the reactor. However, the light penetration depth is so low (e.g., 99 % of the light is absorbed after 0.49 mm at 2.5 g L^−1^ catalyst loading), that even on the edges of the reactor, this “hot zone” where most of the light is absorbed is similar to the center. As an approximation to avoid complex numerical simulations, we therefore employed a virtual volume neutral transformation of the reactor geometry to a rectangular shape with the dimensions 2 × 5 × π/2 cm^3^ (W × H × D) for modeling purposes. With the corresponding virtual optical path length *d* = 1.57 cm and an extinction coefficient of ϵ = 16.4 L g^−1^ cm, this yields an optical density of α = ϵ · *d* · *ln*(10) = 59.3 L g^−1^.

(2) $$\begin{array}{r} \langle r\rangle =\frac{k^{*}}{\alpha }\cdot ln\left(\frac{\phi \cdot q_{p}\cdot \alpha \cdot c_{pc}}{k^{*}\cdot c_{pc}+k_{r}}+1\right)=k_{nb} \end{array}$$

**Figure 6:**
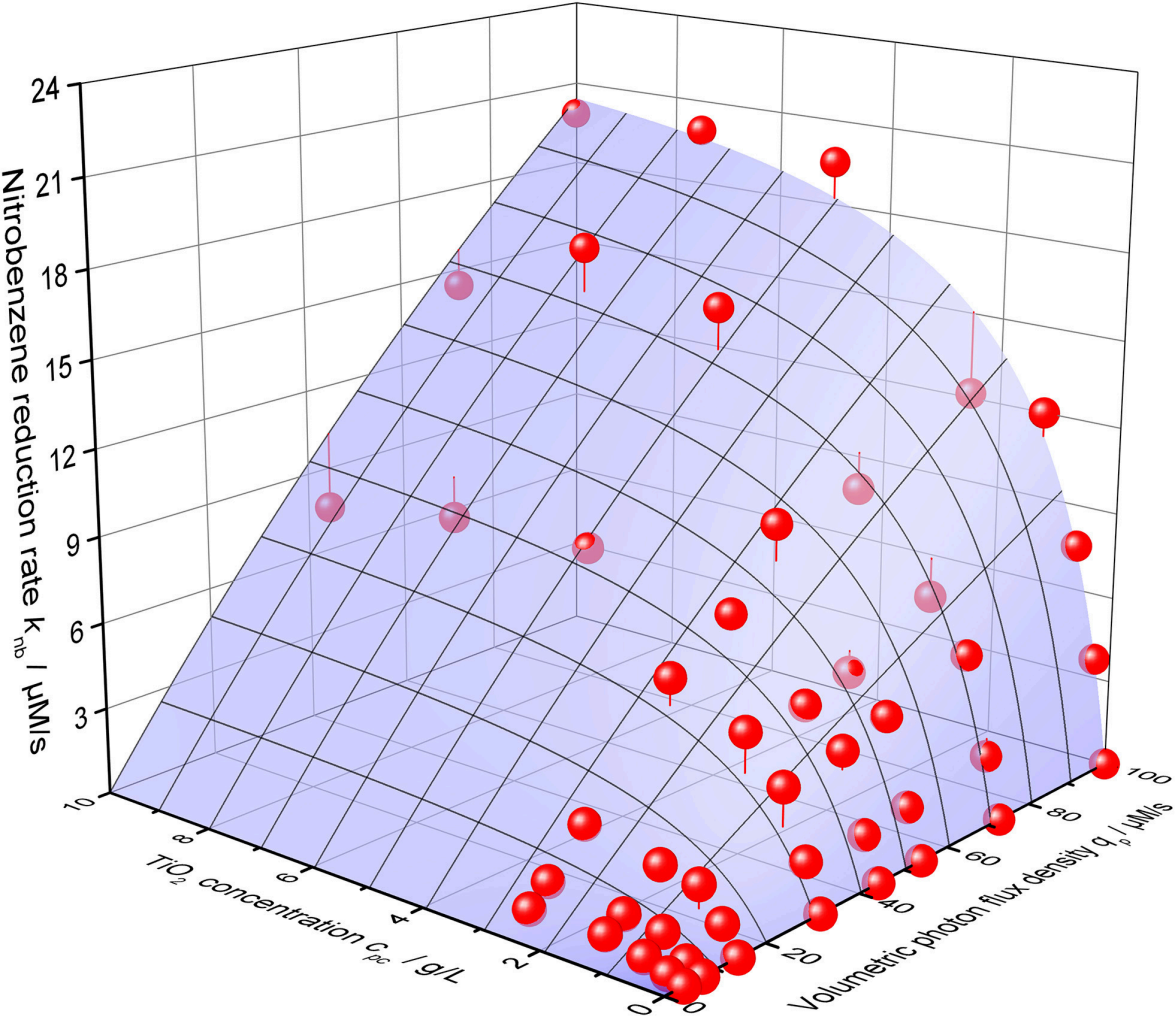
3D-Plot of the nitrobenzene reduction rate *k*_*nb*_ as a function of both the volumetric photon flux density *q*_*p*_ and the photocatalyst concentrations *c*_*pc*_. The blue surface shows the best calculated fit according to Equation 2 with the parameters α = 59.3 L g^−1^, k^*^ = 2558 µmol s^−1^ g, ϕ = 30.7% and *k*_*r*_ = 4483 µM s^−1^ (reaction conditions: 10 mM NB concentration, 25 °C).

Using this equation, the photocatalytic nitrobenzene reduction rates can be modeled with striking precision, *cf*. Figure 6. The corresponding parameters are maximum reaction rate per catalyst mass k^*^ = 2558 µmols^−1^ g^−1^, intrinsic quantum yield *ϕ* = 30.7% and recombination rate *k*_*r*_ = 4483 µM s^−1^.

Since the complete reduction of nitrobenzene to aniline requires six electrons, these results indicate that given sufficiently fast kinetics, apparent quantum yields (AQY) of up to 184 % (6 × ϕ) should be possible for this reaction. In fact, apparent quantum yields of up to 142 % were already experimentally observed in this study (at *c*_*pc*_ = 12.5 g L^−1^, *q*_*p*_ = 44.31 µM s ^−1^, *T* = 25°C). Many of the data points obtained in this study, particularly for higher catalyst concentrations, show apparent quantum yields well over 100 %. These higher than unity quantum yields can be readily explained by the so-called current doubling effect (Hykaway et al., 1986; Schneider and Bahnemann, 2013). The primarily formed ethanol radicals are strong reductants and could reduce nitrobenzene or inject an electron into TiO_2_ which is then used to reduce nitrobenzene (Schwarz and Dodson, 1989). Either way, one photon results in the transfer of two electrons from ethanol to nitrobenzene in this mechanism, which increases the theoretically possible quantum yield to 200 %.

Even considering the current doubling effect, the apparent quantum yields obtained herein are remarkably high and constitute up to 71 % of the theoretical maximum. Using the above mentioned maximum efficiency from the model as reference, ≥21 % efficiency is lost due to kinetic inefficiencies and only 8 % are lost due to photoabsorber inefficiency, i.e., bulk recombination of charge carriers before they reach reactive centers on the surface. Considering that reflection losses (back-scattering from the front window as well as scattering-out of the reaction vessel sides) are not considered at all in this model, the actual incident photon flux is likely lower than assumed here and this would further increase the actual quantum yields. Therefore, the high efficiencies reported herein are likely underestimates and should only be taken as a lower limit (Burek et al., 2019a).

This shows that contrary to popular opinion, heterogeneous photocatalytic transformations can be extremely efficient even with ordinary photocatalysts. Our results disprove the often stated opinion that pristine titania materials suffers from high recombination losses. The processes of light harvesting, charge separation and trapping at surface sites appear to be very efficient with a estimated combined efficiency of 92 %. However, kinetic inefficiencies lead to additional losses which manifest as recombination since the surface trapped charges cannot be transferred to the substrate molecules fast enough. Therefore, it might appear at first glace that recombination is the reason for an observed low efficiency when in fact it is only the symptom of insufficient kinetics.

**Figure 7:**
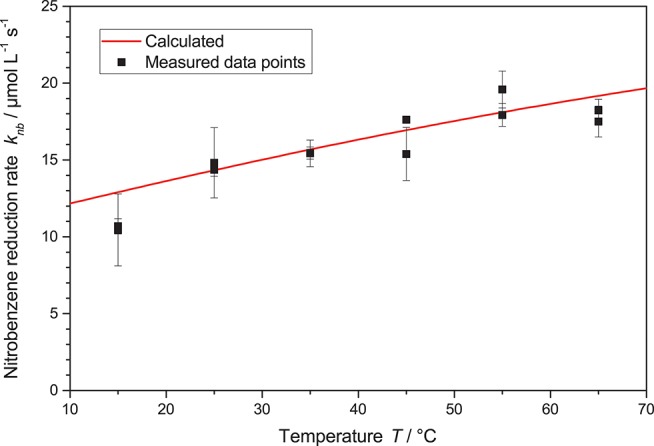
The nitrobenzene reduction rate *k*_*nb*_ in dependence of the temperature *T*. Shown is also the calculated rate according to Equations 2 and 3 with the parameters *A* = 621 mmol s^−1^ g and *E*_*A*_ = 13.6 kJ mol^−1^ (reaction conditions: 2.5 g L^−1^ TiO_2_, 10 mM NB concentration, *q*_*p*_ = 100 µM s^−1^).

Another important parameter of the reaction is the temperature. Traditionally, this is a parameter that has in the past been mostly neglected in heterogeneous photocatalysis. The rationale behind this seems to be that the reaction is initiated by the massive energy provided by the photons, so room temperature is sufficient to drive the reactions (Herrmann, 1999; Malato et al., 2009). Yet, there are some publications which clearly show a temperature dependence of the photocatalytic reactions, we also recently reported positive effects of temperature increase on the photocatalytic hydrogen peroxide formation (Burek et al., 2019a). Therefore, in this study also experiments in the temperature range of 15 °C to 65 °C were performed, higher temperatures were not explored due to the proximity to the boiling point of the solvent ethanol at 78 °C. Generally, the nitrobenzene reduction rate gradually increased with the temperature from 10.6 μM s^−1^ up to 18 μM s^−1^, *cf*. Figure 7. This behavior can be fit well by modulating the reaction rate constant k^*^ in Equation 2 using a classical Arrhenius-approach, Equation 3.

(3) $$\begin{array}{r} k^{*}=A\cdot e^{\frac{-E_{A}}{R\cdot T}} \end{array}$$

The best fit here yields a pre-exponential factor of *A* = 621 mmol s^−1^ g^−1^ and an activation energy of *E*_*A*_ = 13.6 kJ mol^−1^. It is noteworthy that careful analysis of the kinetic model with the obtained parameters reveals that the full temperature effect only becomes apparent at high photon flux, when the requirements on the kinetic constant become so high that they can no longer be met at lower temperatures. When varying the temperature at low reaction rates, i.e., low light intensity, the effect becomes negligible and one might erroneously come to the conclusion that the reaction is insensitive to temperature.

These results indicate that while the activation energy is low in comparison to classical heterogeneous catalytic reactions, even a mild temperature increase can still significantly contribute to better kinetics and improve the overall reaction efficiency. It also appears very advantageous that higher temperatures particularly benefit high reaction rates as these reactions are typically exothermic, either on their own or at least when factoring in photon conversion. For instance, the present reaction features a heat of reaction (calculated from heats of formation) of −296 kJ mol^−1^, not even factoring in the photon energy of at least 984 kJ mol^−1^ (at 365 nm and perfect efficiency of 200 %) (Haynes, 2010). Therefore, the high temperatures necessary for an efficient reaction at high photon flux will inevitably be generated by the reaction anyway, rendering additional heating superfluous.

On top of the nitrobenzene reduction rate, also the corresponding oxidation rate, i.e., formation rate of acetaldehyde, was studied. To properly account for the follow-up reactions, the formation rate of acetaldehyde equivalents, i.e., the sum of acetaldehyde and all follow-up products which contain it, was taken as the basis for kinetic analysis and again the initial part was subjected to linear regression. The resulting acetaldehyde formation rates are shown in Figure S1. Theoretically, this rate should be exactly 3 times as high as the nitrobenzene reduction rate. However, analysis shows that this ratio is in the range from 1 to 1.5 for most of the individual experiments, *cf*. Figure S2. When the data is fitted with the kinetic model (Equation 2) and only the quantum yield is allowed to change from the already obtained parameters, again an excellent fit (*cf*. Figure S3) is obtained with an intrinsic quantum yield of ϕ_*aa*_ = 42.9%, 1.4 times the value of the nitrobenzene reduction. The fact that the observed amounts of acetaldehyde are lower than the expected ratio of three equivalents is likely due to overoxidation. The oxidation rates of ethanol and acetaldehyde over illuminated TiO_2_ are approximately equal so it would not be unlikely that a significant portion of the acetaldehyde is further oxidized to acetate, and eventually, CO_2_ (Burek et al., 2019b). Since acetaldehyde oxidation also liberates two electrons, every molecule of acetaldehyde that is further oxidized replaces one molecule of ethanol. So if a fifth of the acetaldehyde is over-oxidized, already only 2 equivalents of acetaldehyde would be observed. Since the complete oxidation of ethanol liberates 12 electrons, theoretically even half an equivalent of ethanol would be sufficient for complete nitrobenzene reduction. Overall, these results indicate that 36 % of the acetaldehyde are over-oxidized to acetate, assuming that the latter is likely not further oxidized on account of its significantly lower reaction rate (Burek et al., 2019b).

At first glace, it appears strange that the high quantum yields of this reaction have not been reported yet even though it was already studied for several decades. The reason likely lies in the fact that the vast majority of studies have actually not determined the quantum yield. In some cases, the efficiency can be estimated from the data given, Table 1 lists the principal parameters from all major studies on the reaction. It is obvious that the reported or in most cases inferred apparent quantum yields are quite high (>30 % in most cases), supporting our findings. Yet, despite two studies by Selvam et al. which yield of an improbable high apparent quantum yield more than 900 %, likely due to an incorrect determination of the photon flux which appears unrealistically low, the reported numbers are still well below ours. One reason for this could be the often inaccurate (or missing) determination of the incident photon flux which can account for some of the deviation. In most cases, this is only measured using physical probes (measuring irradiance) at approximately the position of the reactor and not inside the actual reaction medium. Another possible explanation is the lack of variation in the studied parameters, so only one or few conditions which were not optimized have been tested.

**Table 1:** An overview of reports for the photocatalytic reduction of nitroaromatic compounds in alcoholic solution.

**Nitroarene**	**Alcohol**	**Photocatalyst**	**q_*p*_ / μM/s**	**c_*pc*_/ g L^−1^**	**T / °C**	**STY / mM h^−1^**	**AQY / %**	**References**
Nitrobenzene (10 mM)	Ethanol	TiO_2_ (P25)	99.9	12.5	25	74.3	124	This study
			44.3	12.5	25	37.6	142	This study
Nitrobenzene (25 mM)	Ethanol	Ag/Au-loaded TiO_2_	1.4	2.0	30	7.9	(956)	Selvam and Swaminathan, 2011a,b
Nitrobenzene (25 mM)	Ethanol	N-doped TiO_2_	1.4	2.0	30	11.3	(1358)	Selvam and Swaminathan, 2012
5-Nitro-*m*-Xylene (10 mM)	Ethanol	TiO_2_ (UV100)	25.3	2.5	n/a	9.0	59	Hakki et al., 2009
*m*-Nitrotoluene (10 mM)	Ethanol	TiO_2_ (P25)	93.6	2.5	25	17.0	30	Hakki, 2013
*m*-Nitrotoluene (10 mM)	Ethanol	TiO_2_ (UV100)	31.2	2.5	n/a	15.0	80	Hakki et al., 2013a
Nitrobenzene (10 mM)	2-Propanol	TiO_2_ (JRC-TiO-6)	8.8	1.0	30	3.1	59	Shiraishi et al., 2012
Nitrobenzene (10 mM)	2-Propanol	TiO_2_ (Rutile)	8.8	1.0	30	3.5	65	Shiraishi et al., 2013
		TiO_2_ (P25)	8.8	1.0	30	2.0	38	Shiraishi et al., 2013
Nitrobenzene (10 mM)	2-Propanol	TiO_2_ (MT-150A)	n/a	10.0	25	76.8	n/a	Imamura et al., 2013
		TiO_2_ (MT-150A)	n/a	10.0	25	n/a	89	Imamura et al., 2013
Nitrobenzene (10 mM)	2-Propanol	Ag/Au-loaded TiO_2_	346.8 (vis)	10.0	25	2.6	1	Tanaka et al., 2013
Nitrobenzene (1.1 mM) in H_2_O	Methanol	Ag-loaded TiO_2_	9.3	1.0	n/a	0.6	11	Tada et al., 2004
Nitrobenzene (167 mM)	2-Propanol	Ag-Cu@ZrO_2_	214.3	16.7	60	3.6	3	Liu et al., 2016

*Shown are the volumetric photon flux q_p_, catalyst concentration c_pc_, temperature T, space-time-yield (STY) and apparent quantum yield (AQY)*.

The space time yield (STY) obtained in the present study (up to 74.3 mM h^−1^ equaling 6.91 g L^−1^ h) is also much higher than most of the values reported up to today. Only one report from Imamura *et al*. shows a similarly high productivity (Imamura et al., 2013). The same authors also report quite a high apparent quantum yield of 89 %, albeit not under the same conditions (Imamura et al., 2013). The differences to our study are explainable by the different choices of alcohol and photocatalyst.

Even though the space-time yields achieved in this report are extremely high for a heterogeneous photocatalytic reaction and amongst the highest reported yet, they are still 1-2 orders of magnitude below what is typically desired in industrial applications (M h^−1^). However, the limiting factor in our case was mainly the light intensity. If the kinetic model is extrapolated to higher light intensities, a STY of 0.27 M h^−1^ should be possible while still maintaining a good apparent quantum yield of 100 % (at *c*_*pc*_ = 12.5 g L^−1^, *q*_*p*_ = 443.56 µM s^−1^, *T* = 65°C). After that point, the efficiency sharply drops so that a STY of 1 M h^−1^ is only possible with extremely high (technically unfeasible) light intensity and with a rather low apparent quantum yield of 16 % (at *c*_*pc*_ = 12.5 g L^−1^, *q*_*p*_ = 10435.56 µM s^−1^, *T* = 65°C). If such high productivities are desired, the intrinsic kinetics of the photocatalyst (k^*^) need to be improved, e.g., by using (noble) metal co-catalysts or alternative reactor concepts with less pronounced light gradients such as internal illumination need to be employed (Burek et al., 2017).

Nonetheless this highlights that heterogeneous photocatalytic reactions have the potential to be immensely intensified and can be both very productive and efficient at the same time. Also, considering the high quantum yield and that the efficiency of LEDs is already at up to 80 % today, the energy efficiency of reactions such as the present one can be very high. This pretty much reduces the challenge of industrial implementation of these reactions to finding suitable and scalable photoreactor concepts. However, recently, much progress has also been made in that area, for instance with photomicroreactors or internal illumination (Burek et al., 2017; Noël, 2017).

## 4. Conclusions

The reaction parameters governing the reaction rate and efficiency of the photocatalytic reduction of nitrobenzene in ethanol were systematically investigated. The effects of light intensity, catalyst concentration, initial concentration and temperature were studied under more than 50 different conditions and accurately described with an appropriate kinetic model. The results show that the efficiency of the reaction is extremely high and only small losses (≥29 %) attributable to catalyst and kinetic inefficiency are observed. Under optimized conditions, apparent quantum yields of up to 142 % were observed, which are, to the best of our knowledge, by far the highest numbers reported for any heterogeneous photocatalytic synthesis.

Particularly interesting is the fact that high efficiencies were also obtained at high reaction rates of up to 74.3 mM h^−1^, extrapolation of the kinetic model to even higher light intensity suggest that up to 0.27 M h^−1^ should be possible for this reaction with reasonably high efficiency. After that point, improvements in either the catalyst or the reactor are required for further intensification.

Overall these results demonstrate that heterogeneous photocatalytic reactions can be very efficient and productive at the same time and may therefore present a powerful tool in synthetic organic chemistry. However, reaching these high efficiencies requires a careful holistic analysis and optimization of the reaction kinetics with respect to all relevant reaction parameters. Given the numbers attained herein, even industrial implementation of this reaction appears feasible in future.

## Data Availability

All datasets generated for this study are included in the manuscript and/or the Supplementary Files.

## Author Contributions

The manuscript was written by JP and JB. JP and BB planned and performed the experiments and did the analysis. The kinetic modeling was done by JB.

### Conflict of Interest Statement

The authors declare that the research was conducted in the absence of any commercial or financial relationships that could be construed as a potential conflict of interest.
