# Supplementary material for: Kinetics and Optimization of the Photocatalytic Reduction of Nitrobenzene
Source: Front Chem. 2019 Apr 24;7:289. doi: 10.3389/fchem.2019.00289 (PMC6491869; doi:10.3389/fchem.2019.00289)
Supplement: Supplementary file 1 [file Data_Sheet_1.pdf]

Supplementary Material for "Kinetics and Optimization of the Photocatalytic Reduction of Nitrobenzene"

The original article is available at http://dx.doi.org/10.3389/fchem.2019.00289

1 SUPPLEMENTARY TABLES AND FIGURES

1.1 Figures

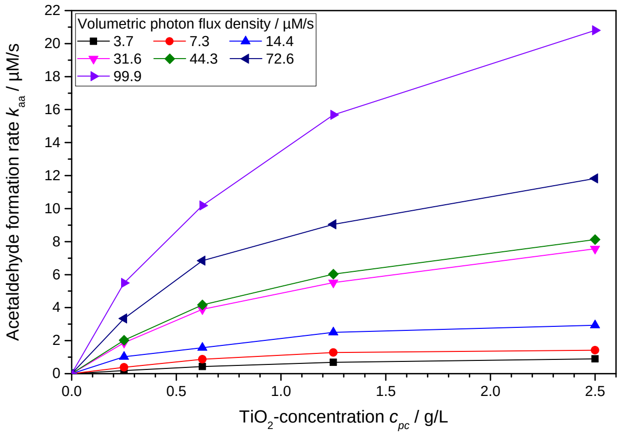

Figure S1. The total acetaldehyde (and acetaldehyde containing compounds) formation rate k_{aa} in dependence of the photocatalyst concentration c_{pc} and volumetric photon flux density q_p (reaction conditions: 10 mM NB concentration, 25 °C).

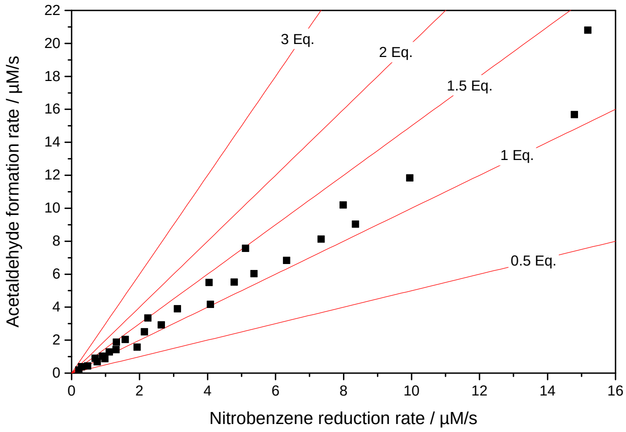

Figure S2. The acetaldehyde formation rate versus the nitrobenzene reduction rate. Plotted are also lines representing 0.5 to 3 equivalents (reaction conditions: 10 mM NB concentration, 25 °C).

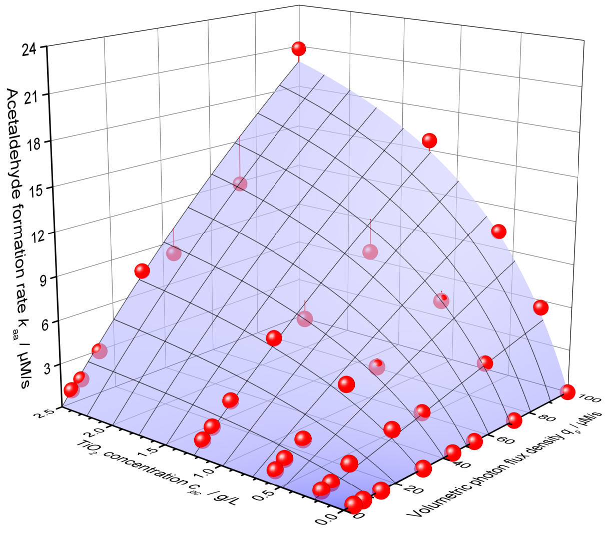

Figure S3. 3D-Plot of the nitrobenzene reduction rate k_{nb} as a function of both the volumetric photon flux density q_p and the photocatalyst concentrations c_{pc}. The blue surface shows the best calculated fit according to eqn. 2 with the parameters $\alpha = 59.3\,\mathrm{L\,g^{-1}}$, $k^* = 2558\,\mathrm{\mu mol\,s^{-1}\,g^{-1}}$, $\phi = 42.9\,\%$ and $k_r = 4483\,\mathrm{\mu M\,s^{-1}}$ (reaction conditions: 10 mM NB concentration, 25 °C).

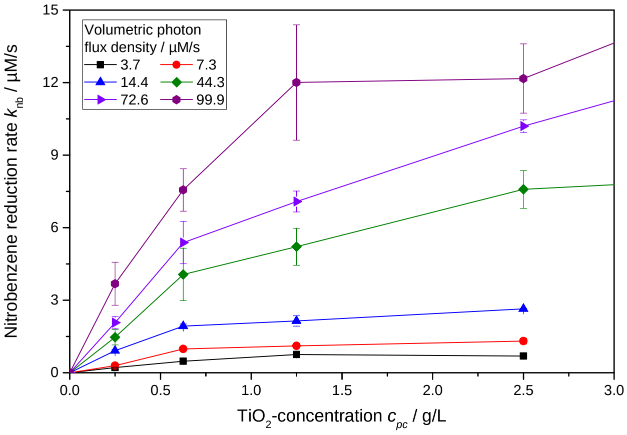

Figure S4. Dependence of the nitrobenzene reduction rate k_{nb} on the photocatalyst concentration c_{pc} for different light intensities (reaction conditions: 10 mM NB concentration, 25 °C), zoomed-in version of Figure 5.
